# Nutritional Challenges and Strategies in Obese Critically Ill Patients with Gynecological Cancer: A Narrative Review

**DOI:** 10.3390/nu18121905

**Published:** 2026-06-12

**Authors:** Maria Fanaki, Dimitrios Haidopoulos, Dimitrios Efthimios Vlachos, Vasileios Lygizos, Antonia Varthaliti, Vasileios Pergialiotis, Georgios Daskalakis, Nikolaos Thomakos

**Affiliations:** First Department of Obstetrics and Gynecology, Division of Gynecologic Oncology, “Alexandra” General Hospital, National and Kapodistrian University of Athens, 11528 Athens, Greece; dimitrioshaidopoulos@gmail.com (D.H.); vlachosdg@gmail.com (D.E.V.); vlygizos@yahoo.com (V.L.); antonia.varthaliti@hotmail.com (A.V.); pergialiotis@yahoo.com (V.P.); gdaskalakis@yahoo.com (G.D.); thomakir@hotmail.com (N.T.)

**Keywords:** obesity, critically ill patients, nutritional support, immunonutrition, enteral nutrition, parenteral nutrition

## Abstract

Critically ill obese patients with gynecological cancer represent a high-risk population with complex nutritional needs. Although excess adiposity may suggest adequate energy reserves, it often conceals sarcopenia, micronutrient deficiencies, and functional malnutrition, contributing to impaired wound healing, immune dysfunction, prolonged mechanical ventilation, increased susceptibility to infections, and adverse oncologic outcomes. Obesity-associated inflammation, insulin resistance, and tumor-driven catabolism further exacerbate metabolic stress and complicate nutritional management in the intensive care setting. Accurate nutritional assessment requires a multimodal approach incorporating body composition analysis, functional measures, and laboratory parameters, as conventional indices such as body mass index may underestimate nutritional risk. Nutritional support should be individualized and may include early enteral nutrition to preserve gut integrity, supplemental or total parenteral nutrition when gastrointestinal function is compromised, high-protein regimens, and targeted micronutrient replacement. Immunonutrition, including arginine, glutamine, omega-3 fatty acids, and nucleotides, has emerged as a promising strategy to modulate inflammation, enhance immune function, and support tissue repair. This narrative review summarizes current evidence regarding obesity-related metabolic dysfunction, nutritional assessment, enteral and parenteral nutrition, and immunonutrition in obese critically ill patients with gynecological cancer. It highlights the challenges associated with sarcopenic obesity and hidden malnutrition while providing a clinically relevant overview for intensivists, gynecologic oncologists, surgeons, and nutrition specialists. Early recognition of nutritional risk and implementation of individualized multimodal nutritional strategies may improve recovery and clinical outcomes. However, high-quality ICU-specific studies remain limited, and further prospective research is needed to establish evidence-based nutritional protocols and evaluate their impact on survival, treatment tolerance, and quality of life in this vulnerable population.

## 1. Introduction

The prevalence of critically ill patients with gynecological cancers admitted to intensive care units shows considerable variation across studies and clinical settings [1,2]. Ovarian cancer is the most frequently observed malignancy, followed by endometrial carcinoma [3]. Patients requiring ICU care commonly present with hemodynamic instability [4] and respiratory complications, alongside higher Charlson Comorbidity Index (CCI) and lower serum albumin levels [5]. In parallel, obesity has emerged as a significant comorbidity in these patients, further influencing disease progression and creating additional challenges for nutritional support in the critical care setting.

Critically ill obese patients with gynecological cancer face unique nutritional challenges. While excess adiposity may suggest sufficient or even excessive nutritional reserves, it frequently masks underlying malnutrition, sarcopenia, and micronutrient deficiencies. These “hidden” nutritional deficits contribute to impaired wound healing, immune dysfunction, prolonged mechanical ventilation, and increased susceptibility to infections, and eventually worse overall survival and disease-free survival [6,7]. Moreover, tumor-driven catabolism, systemic inflammation, and the metabolic stress of critical illness amplify nutrient requirements and complicate the delivery of effective nutritional support [8].

Nutritional management in this population requires careful assessment and individualized strategies. Both enteral and parenteral nutrition play essential roles, complemented by high-protein regimens and targeted micronutrient supplementation [9]. Immunonutrition—incorporating nutrients such as arginine, glutamine, and omega-3 fatty acids—has emerged as a promising adjunct to modulate inflammation, enhance immune competence, and support tissue repair [10].

This narrative review summarizes current evidence on the pathophysiology, clinical impact, and nutritional management of obese critically ill patients with gynecologic cancer. It highlights the interplay between obesity, sarcopenic malnutrition, and metabolic stress to support evidence-based nutritional strategies in this high-risk population. Where gynecologic-specific data are limited, findings from broader oncology or critical care literature are selectively discussed and contextualized to gynecologic malignancies based on shared pathophysiological and perioperative characteristics.

## 2. Materials and Methods

This manuscript is a narrative review synthesizing clinically relevant evidence on nutritional challenges and strategies in obese critically ill patients with gynecologic cancer, a population for whom direct ICU-specific data remain limited. The narrative approach was intentionally selected to allow integration of evidence from gynecologic oncology, mixed oncology cohorts, and general critical care nutrition literature, and to contextualize these findings based on shared pathophysiological mechanisms and gynecologic cancer-specific disease and surgical characteristics. No formal systematic review methodology, risk-of-bias assessment, or quantitative meta-analysis was applied.

### 2.1. Literature Search Strategy

A thorough literature search was performed utilizing electronic databases such as PubMed, Scopus, and Google Scholar. The search parameters were restricted to English-language publications released between July 1975 and May 2025. Keywords and MeSH terms used in the search included: “obesity”, “critically ill patients”, “nutritional support”, “immunonutrition”, “enteral nutrition”, “parenteral nutrition”, “ovarian cancer”, “endometrial cancer”, “cervical cancer”, and “gynecological cancer”. Representative Boolean search strategies included combinations such as: (“obesity” OR “sarcopenic obesity”) AND (“gynecological cancer” OR “ovarian cancer” OR “endometrial cancer” OR “cervical cancer”) AND (“critical illness” OR “ICU”) AND (“enteral nutrition” OR “parenteral nutrition” OR “immunonutrition” OR “nutritional support”). Search syntax was adapted according to the indexing requirements of each database.

PubMed and Scopus were selected to capture peer-reviewed biomedical and clinical nutrition literature, while Google Scholar was included to identify relevant guidelines, consensus statements, and gray literature. This approach is consistent with the narrative review methodology, which aims to synthesize clinically meaningful evidence rather than provide an exhaustive systematic appraisal.

### 2.2. Study Selection and Eligibility Criteria

Inclusion criteria encompassed original research articles, systematic reviews, meta-analyses, and clinical guidelines addressing the pathophysiology, clinical consequences, assessment, and nutritional management of obese gynecological cancer patients in critical care settings. Conversely, the exclusion criteria encompassed case reports, editorials, letters to the editor, and studies solely addressing pediatric populations, non-gynecological cancer cases, or non-surgical scenarios.

### 2.3. Study Screening and Data Extraction

The literature search yielded approximately 424 records across the selected databases. After removal of duplicates, titles and abstracts were screened independently by two reviewers (MF and VP), resulting in the exclusion of 320 records, primarily because they did not address gynecologic malignancies, obesity, critical illness, or nutritional interventions. Full texts of 104 articles were subsequently assessed for eligibility, of which 8 were excluded due to lack of relevance to the study population or outcomes of interest. Ultimately, 96 studies were included in the narrative synthesis. A PRISMA flow diagram was not included, as the review was not designed as a systematic review. Data were extracted regarding metabolic alterations, sarcopenic obesity, micronutrient deficiencies, nutritional assessment tools, and outcomes associated with enteral, parenteral, and immunonutrition strategies in obese gynecological cancer patients.

### 2.4. Evidence Synthesis and Interpretation

Evidence was interpreted through narrative synthesis, acknowledging heterogeneity across study designs, populations, and outcomes. Given the heterogeneity and limited availability of ICU-specific gynecologic oncology studies, evidence was prioritized according to clinical relevance, with preference given to gynecologic oncology populations, followed by mixed oncology and general ICU cohorts when direct evidence was unavailable. Quantitative findings were summarized descriptively where appropriate. Guidance from professional societies, including ESPEN and ASPEN, was incorporated to enhance clinical applicability, particularly where gynecologic oncology-specific ICU data were unavailable.

Priority was given to gynecologic oncology-specific studies, followed by evidence from mixed oncology cohorts and general ICU populations when disease-specific data were lacking. Findings derived from indirect populations are explicitly identified as such in the text and tables. Given the narrative design, the strength of evidence was interpreted qualitatively rather than formally graded.

## 3. Nutritional Challenges in Obese Critically Ill Patients with Gynecological Cancer

### 3.1. Specific Considerations for Obese Critically Ill Gynecologic Cancer Patients

The differentiation between general oncology patients and obese critically ill gynecologic cancer patients arises from the convergence of obesity-specific critical care recommendations and gynecologic cancer-related clinical features, rather than from entirely distinct nutritional physiology. Current critical care guidelines from ASPEN and ESPEN recommend hypocaloric, high-protein feeding strategies for obese critically ill patients to avoid overfeeding while preserving lean body mass—an approach not routinely emphasized in general oncology nutrition guidance [11,12,13,14,15]. Obesity profoundly alters metabolic responses during critical illness, challenging the traditional assumption that excess adiposity confers nutritional reserve. As highlighted by Nelson and Van Way, critically ill obese patients exhibit significant protein catabolism, insulin resistance, and increased risk of overfeeding-related complications, underscoring the need for hypocaloric, protein-focused nutritional strategies in the ICU [16].

Gynecologic cancers, particularly advanced ovarian and high-risk endometrial cancers, introduce additional complexity. These patients exhibit some of the highest reported rates of malnutrition and sarcopenic obesity, often driven by malignant bowel obstruction, ascites, gastrointestinal dysmotility, and treatment-related toxicity [17]. Extensive cytoreductive surgeries, frequently required in ovarian cancer, impose substantial metabolic stress and increase dependence on carefully timed enteral, parenteral, and immunonutrition strategies, often within enhanced recovery after surgery (ERAS) pathways [18].

Furthermore, obese gynecologic oncology patients have elevated risks of surgical complications, cardiovascular comorbidities, insulin resistance, and chemotherapy-related toxicity, necessitating closer monitoring of protein delivery, fluid balance, glycemic control, and micronutrient status in the ICU setting [19]. While many nutritional principles are extrapolated from mixed oncology and critical care populations, their clinical application in this subgroup requires tailored adaptation, underscoring the need for a focused synthesis such as the present review.

### 3.2. Pathophysiology: Inflammation, Insulin Resistance, and Metabolic Dysregulation

Obesity is associated with systemic inflammation, insulin resistance, and altered metabolic states, all of which can affect cancer progression and treatment outcomes in gynecological cancer patients. In obesity, white adipose tissue (WAT) undergoes expansion and becomes an active endocrine organ that secretes a range of pro-inflammatory cytokines, chemokines, and adipokines, all of which contribute to chronic inflammation and immune dysregulation [20]. As WAT outgrows its blood supply, hypoxic conditions develop, leading to adipocyte stress and cell death. This stimulates the release of monocyte chemoattractant protein-1 (MCP-1), attracting macrophages that surround dying fat cells and form crown-like structures (CLSs) [21]. These CLSs create a highly inflammatory environment that impairs normal immune surveillance and promotes tumor progression by supporting cancer cell proliferation and resistance to apoptosis [22]. Additionally, free fatty acids released from damaged adipocytes activate toll-like receptor 4 (TLR4) on macrophages, enhancing the expression of pro-inflammatory mediators such as TNF-α, IL-1β, and COX-2 through NF-κB signaling [23]. This sustained inflammation not only weakens immune function but also fosters a tumor-permissive microenvironment, contributing to the increased aggressiveness and poor prognosis observed in many obesity-related cancers.

Moreover, obesity leads to an imbalance in adipokine secretion, marked by elevated levels of pro-inflammatory adipokines like leptin and decreased levels of anti-inflammatory adipokines such as adiponectin [24]. In addition, obesity, especially central or visceral obesity, is closely associated with insulin resistance (IR), resulting in compensatory hyperinsulinemia and impaired regulation of glucose metabolism [25]. Hyperglycemia further impairs neutrophil function, promoting oxidative stress, elevated inflammatory cytokines, and metabolic dysregulation [26].

In parallel, gynecological cancers are not only localized neoplastic diseases but also systemic inflammatory conditions. The presence of malignancy induces a tumor-associated inflammatory response that significantly contributes to metabolic dysregulation, especially in the setting of obesity and critical illness. Tumor cells secrete a range of proinflammatory cytokines, chemokines, and growth factors into the tumor microenvironment and systemic circulation. These include interleukin-6 (IL-6), interleukin-1β (IL-1β), tumor necrosis factor-alpha (TNF-α), and transforming growth factor-beta (TGF-β). These mediators activate signaling pathways such as NF-κB and JAK/STAT, which promote catabolic processes and suppress anabolic pathways, leading to muscle protein breakdown, anorexia, and impaired glucose and lipid metabolism [27]. In ovarian cancer, for example, high levels of IL-6 correlate with advanced disease stage, cachexia, and poor prognosis [28].

In critically ill obese cancer patients, chronic obesity-associated inflammation may transition into an exaggerated acute hypercatabolic response triggered by surgery, sepsis, or organ dysfunction. Activation of IL-6/JAK-STAT and NF-κB signaling pathways promotes proteolysis, insulin resistance, mitochondrial dysfunction, and impaired muscle protein synthesis, contributing to anabolic resistance and accelerated skeletal muscle loss. Simultaneously, adipose tissue inflammation and adipokine imbalance, characterized by elevated leptin and reduced adiponectin levels, further impair immune regulation and metabolic homeostasis. These alterations contribute to cancer cachexia and sarcopenic obesity by promoting immune suppression, oxidative stress, and progressive depletion of lean body mass despite excess adiposity.

The tumor-associated inflammatory response also disrupts endocrine regulation of appetite and energy balance through alterations in leptin, ghrelin, and hypothalamic signaling. Patients frequently develop cancer-associated anorexia, early satiety due to tumor mass effect [29], and gastrointestinal dysmotility [30], all of which reduce oral intake and contribute to energy deficit. Furthermore, the presence of chronic inflammation in these patients alters the pharmacokinetics of many nutrients and therapeutic agents. For example, hypoalbuminemia induced by IL-6-mediated hepatic reprioritization not only indicates malnutrition but also alters drug binding and distribution [31]. Additionally, inflammatory cytokines impair micronutrient absorption and utilization, leading to deficiencies in zinc, selenium, iron, and vitamin D—all of which are crucial for immune competence and tissue repair [32].

Emerging evidence suggests that alterations in gut microbiota may further contribute to metabolic dysfunction in obese critically ill cancer patients. Obesity is associated with intestinal dysbiosis characterized by reduced microbial diversity, increased intestinal permeability, and activation of pro-inflammatory pathways. In critically ill patients, factors such as broad-spectrum antibiotics, vasopressor therapy, mechanical ventilation, and reduced enteral nutrient delivery may further disrupt microbiome composition. These alterations may promote bacterial translocation, systemic inflammation, insulin resistance, and impaired immune regulation, thereby potentially exacerbating cancer-related metabolic disturbances and malnutrition. Early enteral nutrition may partially preserve gut barrier integrity and microbial homeostasis, whereas prolonged gastrointestinal dysfunction and exclusive parenteral nutrition may further impair microbial diversity. Although evidence specifically focusing on critically ill gynecologic oncology patients remains limited, the interaction between obesity, critical illness, cancer, and the gut microbiome represents an emerging area of research with potential implications for future nutritional interventions.

**Figure 1:**
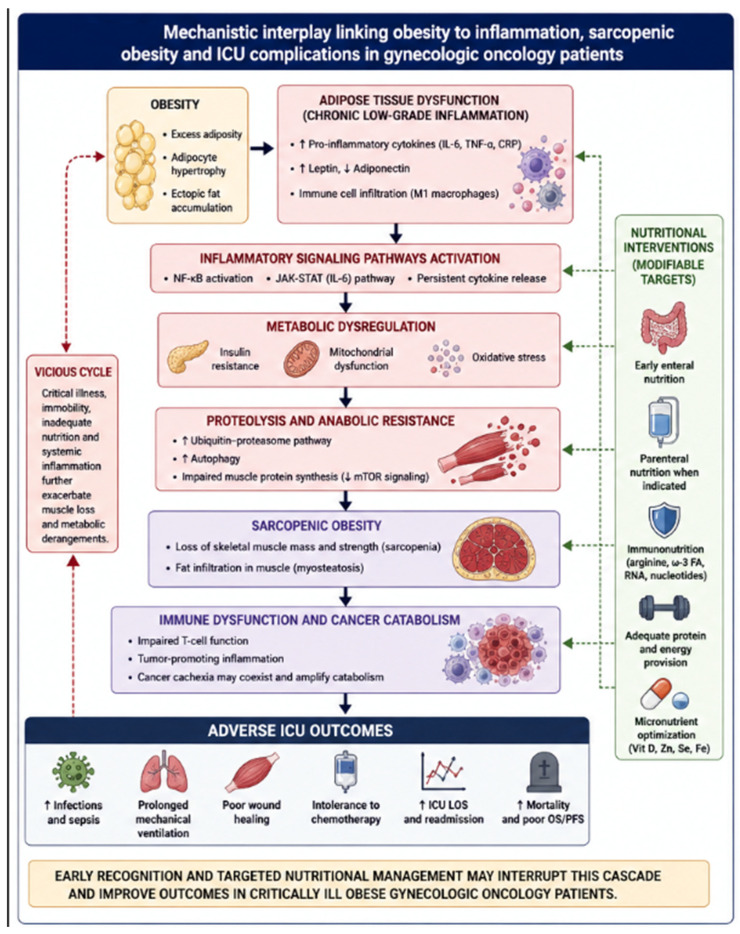
Mechanistic interplay linking obesity to inflammation, sarcopenic obesity, and ICU complications in gynecologic oncology patients.

When overlaid with the inflammation and catabolism of critical illness—such as sepsis, major surgery, or mechanical ventilation—the tumor-induced inflammatory state significantly increases metabolic stress. This results in an exaggerated hypercatabolic state characterized by rapid lean tissue loss and profound insulin resistance. This makes nutritional management particularly challenging, as traditional feeding strategies may be insufficient or even harmful if not appropriately tailored (Figure 1).

### 3.3. Sarcopenic Obesity

In patients with gynecologic cancers—particularly ovarian and endometrial malignancies—sarcopenic obesity represents a clinically relevant phenotype associated with adverse surgical, ICU, and oncologic outcomes. Sarcopenic obesity (SO) is characterized by the combination of obesity, defined by high body fat percentage, and sarcopenia, defined as low skeletal muscle mass accompanied by low muscle function. Its prevalence rates vary depending on population and diagnostic criteria, but may affect up to 15–36% of obese cancer patients in critical care [33], and has been increasingly recognized as a major contributor to adverse clinical outcomes in cancer patients, including those with gynecologic malignancies. According to the ESPEN and EASO Consensus Statement, the diagnosis of SO is performed in two sequential steps. First, skeletal muscle function is evaluated using tests such as hand-grip strength, knee extensor strength adjusted for body mass, or chair-stand tests, with cut-off points validated according to sex, ethnicity, and age. If muscle function is found to be low, the assessment proceeds to the second step, which involves evaluating body composition (Table 1, [34,35,36,37,38,39,40,41,42]). This is primarily achieved using dual-energy X-ray absorptiometry (DXA), with bioelectrical impedance analysis (BIA) as an alternative, and computerized tomography (CT) utilized when available, particularly in patients undergoing CT scans for other clinical reasons [43]. However, universally accepted diagnostic criteria for sarcopenic obesity remain lacking, and substantial variability exists across CT-derived cut-offs, ethnicity-specific thresholds, and functional versus imaging-based definitions.

Sarcopenic obesity in cancer patients results from a complex interplay between skeletal muscle loss and adipose tissue dysfunction, driven by metabolic imbalances such as insulin resistance, inflammation, and hormonal disturbances. Physical inactivity and inadequate nutrition, especially low omega-3 fatty acid intake, impair muscle protein synthesis [44] and promote fat infiltration in muscles (myosteatosis), which further reduces muscle function [45]. Adipose tissue inflammation leads to excessive fatty acid release and lipid accumulation within muscle cells, disrupting glucose uptake and mitochondrial function, causing oxidative stress and muscle degradation [46]. Additionally, increased secretion of pro-inflammatory cytokines and reduced production of anti-inflammatory myokines further promote muscle wasting and fat accumulation [47]. Together, these mechanisms create a vicious cycle that contributes to progressive sarcopenic obesity, tumor progression, and poorer cancer outcomes. Unlike cancer cachexia, which is primarily characterized by severe systemic catabolism and involuntary weight loss, sarcopenic obesity may remain clinically underrecognized because excess adiposity masks progressive skeletal muscle depletion.

### 3.4. Clinical Consequences of Malnutrition Hidden by Obesity in Patients with Gynecological Cancer

The assumption that being obese means having sufficient or excess nutrition has led to overlooked malnutrition in obese individuals—especially those undergoing cancer treatment. In reality, obesity can hide serious problems such as reduced muscle mass, shortages in essential nutrients, and impaired physical function—all of which critically affect patient outcomes. This type of “hidden malnutrition” is especially risky for severely ill patients with gynecologic cancers, who urgently need nutritional support that is often delayed or poorly customized. Conventional nutritional screening methods often rely on weight-based indices such as Body Mass Index (BMI), which cannot distinguish adipose tissue from lean body mass [48]. Consequently, obese patients with advanced ovarian cancer may have significant muscle depletion despite being classified as “normal” or “overweight,” potentially leading to under-recognition of nutritional risk and delayed intervention (Table 2).

Sarcopenia also reduces the body’s capacity to synthesize structural proteins required for tissue repair. In gynecological oncology, extensive debulking surgeries or pelvic exenteration procedures demand optimal healing capacity. Obese patients with underlying malnutrition may experience delayed wound closure, increased rates of wound dehiscence, and surgical site infections [49].

Protein-energy malnutrition also impairs both innate and adaptive immune responses. In obese gynecological cancer patients, systemic inflammation from obesity exacerbates immune dysfunction, increasing their susceptibility to postoperative infections [50,51,52]. In an observational study of endometrial cancer patients nosocomial infections occurred significantly more often in malnourished (52.1%; 25/48) and severely malnourished (42.1%; 8/19) patients compared to those who were well-nourished (25%; 10/40) (*p* = 0.035), and therefore they had a significantly longer stay compared to those without infections (18.6 vs. 10.8 days, *p* < 0.024) [53]. In addition, patients with sarcopenic obesity (SO) had significantly higher hospital costs and a higher rate of readmission within 30 days following gastrectomy for gastric cancer [54]. They also showed a notably higher incidence of postoperative complications—such as abscesses, cardiac issues, and pulmonary problems—after undergoing pancreatoduodenectomy, in comparison to obese patients without sarcopenia [55].

Additionally, undernourished patients with decreased respiratory muscle mass may struggle to wean from ventilatory support. A meta-analysis of 3582 ovarian cancer patients demonstrated that sarcopenic patients presented longer duration of mechanic ventilation support (MD = 1.22; 95% CI 0.39, 2.05) longer ICU stay (MD = 1.31; 95% CI 0.43, 2.19), and overall hospital stay (MD 2.73; 95% CI 0.58, 4.88; *I*^2^ = 98.0%) compared with patients with normal nutritional status [61].

The connection between SO and increased chemotherapy toxicity is thought to stem from the combination of high absolute drug doses and a reduced volume of distribution. Chemotherapy dosing is typically based on body surface area (BSA), and in patients with SO, a higher BSA may lead to larger doses. However, these doses are distributed within and processed by a significantly reduced lean body mass (LBM), which may lead to a greater risk of toxicity due to impaired drug metabolism and clearance [62]. According to the results of a retrospective study of oesophageal cancer patients that received neoadjuvant chemotherapy, sarcopenic obesity had a significantly elevated risk of dose-limiting toxicity (OR = 5.54; 95% CI: 1.12–27.44) [57]. Another study including patients undergoing chemoradiotherapy for cervical cancer demonstrated that those with sarcopenic obesity—defined as low skeletal muscle mass combined with high adiposity—had approximately a fourfold increased risk of chemotherapy toxicity-induced treatment modifications (TIMT), such as delays or interruptions [58].

Although sarcopenia, sarcopenic obesity, cachexia, frailty, and myosteatosis frequently overlap in oncology patients, they represent distinct clinical entities with differing pathophysiological mechanisms and therapeutic implications. Cancer cachexia is primarily characterized by severe systemic catabolism and involuntary weight loss, whereas sarcopenic obesity may remain clinically underrecognized because excess adiposity masks progressive skeletal muscle depletion. Frailty reflects a broader decline in physiological reserve and functional capacity, while myosteatosis refers to pathological fat infiltration within skeletal muscle that impairs muscle quality and metabolic function.

### 3.5. Survival Outcomes and Impact of Nutritional Status in Critically Ill Obese Patients with Gynecological Cancer

Multiple studies have demonstrated that sarcopenic obesity serves as a distinct negative prognostic factor associated with overall survival and progression-free survival in patients with gynecologic malignancies. The concurrent presence of excess adipose tissue and skeletal muscle depletion contributes to increased vulnerability, compromised immune function, and a reduced capacity to endure physiological stressors induced by cancer and its treatment.

In a prospective analysis involving 226 women diagnosed with gynecological cancer, researchers found that having a Body Mass Index (BMI) above 25 kg/m^2^ and a phase angle α below 4.75°, as measured by bioelectrical impedance (a tool used to assess nutritional status), were both strongly linked to serious complications within 30 days following surgery, as well as reduced overall survival rate [59]. Another prospective study of 176 patients with high-grade endometrial cancer showed sarcopenic obesity was associated with reduced overall survival (*p* = 0.048) [60]. A recent meta-analysis including 4136 patients with gynecological cancer revealed a high pooled prevalence of sarcopenia in gynecologic oncology patients at 38.8% (95% CI: 0.49–0.79, *I*^2^ = 96%, *p* < 0.001). In particular, sarcopenia was significantly associated with poorer overall survival in patients in the ICU—several studies reported a markedly lower 5-year OS (e.g., 10.4% vs. 43.4%, *p* < 0.001)—and reduced progression-free survival (PFS), with sarcopenic patients showing PFS as low as 3.5 months compared to up to 29.1 months in non-sarcopenic patients [56].

### 3.6. Micronutrient Deficiencies in Obese Cancer Patients

An often overlooked but clinically significant aspect of malnutrition in obesity is the prevalence of micronutrient deficiencies. Although obese patients present with excess adipose tissue and energy reserves, they are not protected against deficiencies of essential vitamins and minerals. In fact, obesity is frequently associated with micronutrient imbalances due to altered dietary patterns, impaired absorption, chronic inflammation, and sequestration of nutrients within adipose tissue. In gynecological cancer patients, these deficiencies are further compounded by tumor-related catabolism, gastrointestinal symptoms, and the metabolic stress of critical illness. Such deficiencies are of particular concern in critically ill patients, as they can impair immune function, hinder wound healing, and reduce tolerance to cancer treatments [63].

Vitamin D deficiency is especially prevalent in obese populations because vitamin D is fat-soluble and becomes sequestered in adipose tissue, reducing its bioavailability. In cancer patients, low vitamin D levels are associated with impaired immune surveillance, increased susceptibility to infections, and reduced muscle strength, which further contributes to sarcopenia [64]. In gynecological cancers, vitamin D insufficiency has been linked to poorer treatment outcomes, increased bone fragility from estrogen-depleting therapies, and a higher risk of fractures during survivorship [65]. Critically ill patients with vitamin D deficiency also demonstrate higher rates of sepsis and prolonged ICU stays [66].

Iron deficiency is another common problem, stemming from multiple mechanisms in gynecological cancer patients. Poor dietary intake, tumor-related blood loss, and inflammation-driven functional iron deficiency all contribute to anemia of chronic disease. Elevated hepcidin levels during systemic inflammation reduce intestinal iron absorption and trap iron within macrophages, rendering it unavailable for erythropoiesis. This results in anemia, fatigue, reduced exercise tolerance, and diminished response to chemotherapy or radiotherapy. Iron deficiency anemia has also been associated with increased perioperative transfusion requirements, which carry their own risks in surgical oncology [67].

Zinc and selenium deficiencies, though often overlooked, are vital to recovery and survival. Zinc supports cell growth, protein synthesis, and wound healing, but inflammation, poor intake, and altered absorption in obese cancer patients can worsen deficiency [68]. Selenium, crucial for antioxidant defense, protects against oxidative stress; low levels impair immunity, delay healing, and heighten vulnerability to chemotherapy and illness [69]. In gynecologic oncology surgery, inadequate levels of both have been linked to slower recovery and more infections [70].

Addressing micronutrient deficiencies in obese gynecological cancer patients requires a proactive approach, including targeted screening, individualized supplementation, and integration into comprehensive nutrition support plans. For critically ill patients, intravenous or enteral supplementation may be necessary to bypass gastrointestinal limitations and ensure adequate bioavailability [71].

### 3.7. Nutritional Status Assessment and Scoring Tools

Assessing nutritional status in obese gynecological cancer patients presents significant challenges, as conventional measures often fail to capture underlying muscle loss and metabolic compromise. Body mass index (BMI) does not distinguish lean body mass from fat mass, allowing obese patients with severe muscle depletion to appear adequately nourished. This limitation is further compounded by ascites and edema, which frequently distort weight-based assessments in advanced ovarian cancer and critical illness [72]. Moreover, commonly used biochemical markers, including albumin and prealbumin, are strongly influenced by inflammation and the acute-phase response rather than true nutritional status [73]. Finally, sarcopenia often remains hidden in these patients, as significant skeletal muscle loss can only be reliably detected through advanced body composition techniques such as computed tomography (CT), dual-energy X-ray absorptiometry (DXA), or bioelectrical impedance analysis (BIA) (Table 1). These limitations underscore the need for more accurate, multimodal nutritional assessment strategies in this population.

To improve transparency regarding the strength and applicability of the available evidence, Table 3 summarizes key studies supporting the associations between sarcopenia, nutritional interventions, and clinical outcomes in gynecologic oncology and related critical care populations.

### 3.8. ICU-Specific Nutritional Considerations

**Figure 2:**
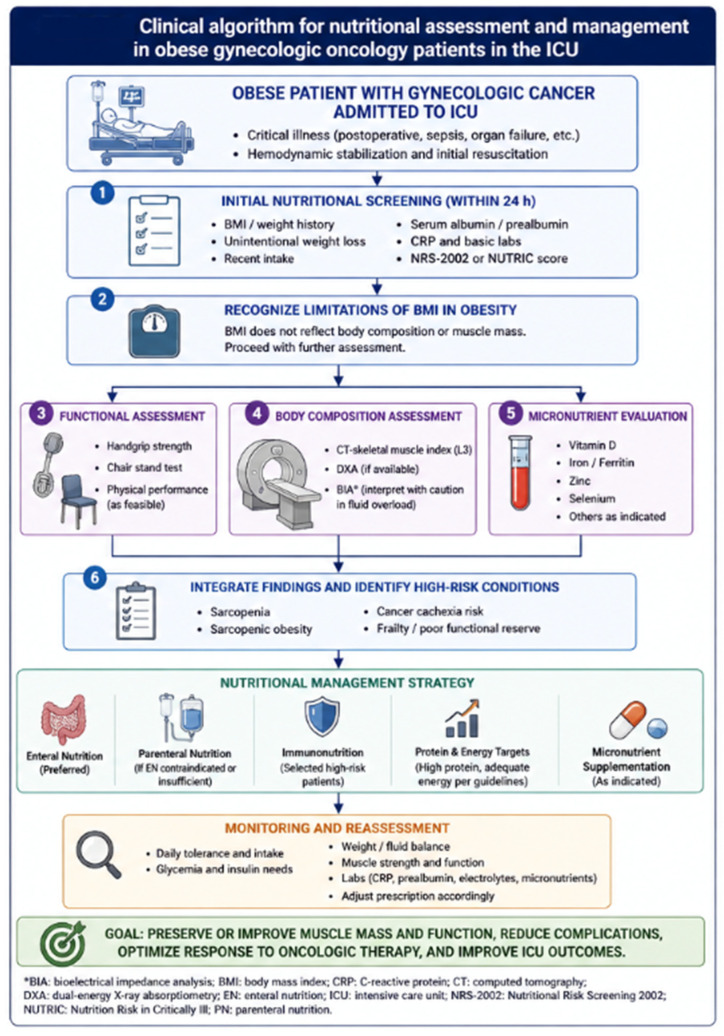
Clinical algorithm for nutritional assessment and management in obese gynecologic oncology patients in the ICU.

ICU-specific nutritional management in obese gynecologic oncology patients presents additional challenges, including altered enteral feeding tolerance during vasopressor support, increased risk of refeeding syndrome, glycemic variability, and severe protein catabolism associated with sepsis and prolonged mechanical ventilation. Indirect calorimetry may improve estimation of energy expenditure when available, while nitrogen balance monitoring can assist in evaluating adequacy of protein delivery during prolonged ICU stays. Careful progression from trophic to full feeding is often required in hemodynamically unstable patients (Figure 2).

## 4. Enteral Nutrition

Enteral nutrition (EN) is widely regarded as the preferred route of feeding in critically ill patients whenever the gastrointestinal tract is functional. For obese gynecological cancer patients, EN provides not only macronutrients and micronutrients but also contributes to the preservation of gut integrity, modulation of immune responses, and attenuation of systemic inflammation—factors that are critically important in this population. Specifically, nutrient delivery via the gut stimulates enterocyte proliferation, supports mucosal barrier function, and reduces bacterial translocation and the risk of sepsis. The intestinal tract serves as a critical barrier against bacterial pathogens and intraluminal toxins, primarily due to the rapid renewal of enterocytes within the epithelial lining, the protective mucus produced by goblet cells, and the extensive presence of lymphoid tissue that constitutes a robust immune defense. Notably, approximately 80% of the body’s immunoglobulin production—predominantly immunoglobulin A (IgA)—is secreted via the gastrointestinal system, which also houses nearly 50% of the total immune cell mass, underscoring its central role in immunological function [75]. EN also promotes the secretion of gastrointestinal hormones and bile acids, supporting digestion and metabolic regulation, reducing the risk of hyperglycemia and hepatic steatosis in the postoperative period. These effects are particularly valuable in gynecological cancer patients who frequently undergo extensive abdominal or pelvic surgery, as maintenance of gut integrity reduces the risk of postoperative infections and supports recovery [76,77].

However, the use of enteral nutrition (EN) in obese cancer patients poses specific challenges, including altered energy requirements, feeding intolerance, and higher risks of complications such as aspiration, delayed gastric emptying, and anastomotic leakage. A key difficulty lies in establishing appropriate caloric and protein targets, as traditional weight-based formulas often result in overfeeding, contributing to hyperglycemia, hepatic steatosis, and increased ventilatory burden. ASPEN recommends initiating supplemental parenteral nutrition (SPN) after six days if enteral nutrition is insufficient, while parenteral nutrition (PN) can be started at any time. Protein intake is advised at 1.2–2.0 g/kg/day, with energy provision of 12–25 kcal/kg/day, particularly within the first 7–10 days [13]. In contrast, ESPEN guidelines advocate for earlier initiation, recommending both SPN and PN within 3–7 days. ESPEN suggests a protein target of 1.3 g/kg/day and advises limiting energy delivery to ≤70% of estimated energy expenditure (EE) during days 1–3, progressing to 80–100% of EE after day 3. Early EN, initiated within 24–48 h, has been shown to attenuate catabolism and improve outcomes in critically ill populations, including oncology patients [12].

Protein is a critical macronutrient in critical illness, supporting wound healing, immune function, and preservation of lean body mass. Current guidelines recommend 1.2–2.0 g/kg/day (SCCM/ASPEN) or approximately 1.3 g/kg/day (ESPEN) [13,78]. Observational studies suggest that achieving higher protein intakes is associated with reduced mortality, whereas meeting caloric targets without adequate protein offers no survival benefit. The timing of protein delivery remains debated: while some studies demonstrate lower mortality with early supplementation in nonseptic, non-overfed patients, others report adverse outcomes with high protein administration in the early ICU phase. In the absence of randomized controlled trials, clinical practice typically applies uniform protein targets across the ICU stay, without adjustment for the initial catabolic phase [79].

Feeding tolerance in obese gynecologic cancer patients may be impaired by several factors, including delayed gastric emptying due to surgery, opioid use, or critical illness; abdominal compartment syndrome or ascites in advanced ovarian cancer, which restricts gastrointestinal motility; and elevated aspiration risk in patients with impaired consciousness or requiring mechanical ventilation. Strategies to optimize tolerance include the use of prokinetic agents (e.g., metoclopramide, erythromycin), elevating the head of the bed to ≥30°, employing continuous rather than bolus feeding, and transitioning to post-pyloric feeding when necessary.

## 5. Parenteral Nutrition

Parenteral nutrition (PN) constitutes an essential modality of nutritional support for gynecological cancer patients, especially patients undergoing major debulking surgeries, who are unable to tolerate or effectively absorb enteral nutrition (EN). Although EN is generally favored due to its physiological advantages, PN becomes indispensable under specific clinical circumstances. These include postoperative gastrointestinal dysfunction following extensive cytoreductive procedures, bowel resections, or complications such as anastomotic leakage and ileus, which frequently impede enteral feeding. Additionally, malignant bowel obstruction—commonly associated with advanced ovarian or endometrial carcinomas presenting with peritoneal carcinomatosis, ascites, and intestinal blockage—renders EN unfeasible. Severe mucositis or enteritis induced by radiation therapy, chemotherapy, or multimodal oncologic treatments may also compromise mucosal integrity and nutrient absorption, necessitating PN. Furthermore, in critically ill patients exhibiting gastrointestinal intolerance—characterized by hemodynamic instability, elevated gastric residual volumes, or recurrent aspiration risk—PN may be required either temporarily or as a supplemental intervention to ensure adequate nutritional delivery [11].

In the context of obesity, PN presents unique challenges. Despite the presence of substantial adipose reserves, obese cancer patients often exhibit sarcopenia, systemic inflammation, and metabolic dysregulation, necessitating individualized PN strategies to avoid both underfeeding and overfeeding. Protein and caloric targets generally follow ASPEN and ESPEN recommendations previously described for critically ill obese patients. ASPEN recommends initiating supplemental parenteral nutrition (SPN) after six days if enteral nutrition is insufficient, while parenteral nutrition (PN) can be started at any time. Protein intake is advised at 1.2–2.0 g/kg/day, with energy provision of 12–25 kcal/kg/day, particularly within the first 7–10 days. In contrast, ESPEN guidelines advocate for earlier initiation, recommending both SPN and PN within 3–7 days. ESPEN suggests a protein target of 1.3 g/kg/day and advises limiting energy delivery to ≤70% of estimated energy expenditure (EE) during days 1–3, progressing to 80–100% of EE after day 3. In gynecological oncology, particularly among patients experiencing postoperative complications or prolonged ileus, early PN has been associated with improved nitrogen balance and reduced infectious morbidity.

Protein provision is particularly critical for preserving lean body mass. Guidelines advocate for ≥2.0 g/kg of ideal body weight per day, with potential escalation to 2.5 g/kg in cases of severe metabolic stress. Even within hypocaloric regimens, prioritizing protein delivery is essential, as high-protein PN has been shown to mitigate negative nitrogen balance in sarcopenic obesity. Intravenous lipid emulsions offer a concentrated energy source while minimizing glucose burden. Mixed-oil emulsions—comprising medium-chain triglycerides, olive oil, or fish oil—are preferred over pure soybean oil due to their reduced proinflammatory properties. Omega-3-enriched emulsions may confer additional benefits by modulating inflammatory pathways, lowering infection rates, and enhancing immune function in oncology patients.

Carbohydrate administration, particularly glucose, should be carefully titrated to prevent hyperglycemia, especially in obese individuals with insulin resistance. Continuous glucose monitoring and insulin infusion protocols may be necessary to maintain glycemic control. Micronutrient deficiencies are common in obese cancer patients, with frequent deficits in vitamin D, iron, zinc, selenium, and magnesium. PN formulations should be customized to address these deficiencies, as inadequate replacement may impair wound healing, immune competence, and overall recovery. For instance, selenium supports antioxidant defenses, while zinc plays a pivotal role in tissue repair following oncologic surgery [80].

Despite its benefits, PN carries specific risks, which are often amplified in obese cancer patients. These include metabolic disturbances such as hyperglycemia, hypertriglyceridemia, and electrolyte imbalances [81], largely attributable to underlying insulin resistance and altered metabolic profiles [82]. Infectious complications are also a concern, as central venous catheters used for PN delivery increase the risk of bloodstream infections, particularly in immunocompromised individuals [83]. Hepatic complications, including steatosis and cholestasis, may arise with long-term PN, with heightened susceptibility in patients with pre-existing nonalcoholic fatty liver disease. Additionally, fluid overload must be carefully managed, especially in individuals with ascites, cardiac dysfunction, or renal impairment [84].

Preventive measures include stringent catheter care, selection of lipid emulsions with lower proinflammatory potential, and rigorous metabolic monitoring. Although high-quality clinical trials specifically targeting obese gynecological cancer patients remain limited, existing evidence supports the role of PN in preserving nutritional status, preventing severe protein-energy malnutrition, and facilitating tolerance to intensive oncologic therapies. Studies involving advanced ovarian cancer with malignant bowel obstruction suggest that PN may extend survival, maintain functional capacity, and enhance quality of life in selected patients [85]. In postoperative oncology settings where EN is not viable, PN has demonstrated efficacy in reducing nitrogen losses, promoting wound healing, and lowering complication rates when appropriately administered [86]. Crucially, optimal outcomes are achieved when PN is integrated into a comprehensive, multidisciplinary nutritional care plan involving dietitians, oncologists, and critical care specialists.

## 6. Immunonutrition

Immunonutrition refers to the targeted use of specific nutrients—such as omega-3 fatty acids, arginine, glutamine, and nucleotides—that exert immunomodulatory, anti-inflammatory, and tissue-reparative effects beyond their caloric and protein contributions. In critically ill gynecological cancer patients who experience profound systemic inflammation, metabolic stress, and immune dysregulation, immunonutrition offers a promising therapeutic adjunct. The rationale is particularly strong in this population: tumor-associated inflammation, surgical trauma, obesity-driven immune dysfunction, and chemotherapy-induced immunosuppression converge to create a state of heightened susceptibility to infection, impaired wound healing, and catabolic muscle loss [87].

Immunonutrition demonstrates the greatest benefit when initiated early—ideally 5–7 days preoperatively and continued into the postoperative or ICU period, or at the start of enteral or parenteral nutrition in critically ill patients. Key substrates include arginine, which supports T-cell proliferation, nitric oxide synthesis, and collagen deposition, thereby enhancing wound healing and immune function; however, caution is warranted in sepsis due to potential nitric oxide-mediated vasodilation [88]. Although arginine may enhance immune function and wound healing, concerns persist regarding potential hemodynamic effects in severe sepsis due to nitric oxide-mediated vasodilation, and current evidence remains inconsistent. Omega-3 fatty acids (EPA, DHA) modulate inflammatory pathways by replacing arachidonic acid in cell membranes, reducing proinflammatory eicosanoids, and attenuating cytokine-driven responses via NF-κB downregulation [89]. Glutamine, a principal energy source for enterocytes and lymphocytes, is frequently depleted in critically ill and cancer patients, and its deficiency has been associated with impaired gut barrier integrity, immune dysfunction, and increased morbidity [90]. Nucleotides, though less extensively studied, contribute to DNA repair, lymphocyte proliferation, and antibody synthesis, which are of particular relevance in patients undergoing chemotherapy or radiotherapy [91].

Although immunonutrition has demonstrated potential in reducing postoperative complications and improving recovery, several challenges hinder its routine clinical adoption. Patient compliance remains a major barrier, particularly in the postoperative phase, where nausea, vomiting, appetite loss, and lack of motivation significantly reduce adherence. Tailored formulations, alternative delivery routes, and structured nutrition support programs, combined with patient education and food literacy interventions, are essential to improve tolerance and adherence [92]. Cost is another limitation, as immune-enhancing formulations are more expensive than standard options; however, evidence indicates that upfront expenses can be offset by long-term savings through reduced infection rates, shorter hospital stays, and fewer readmissions. Variability in formulations across clinical trials further complicates interpretation and standardization, contributing to inconsistent outcomes and clinician hesitancy [93]. Finally, conflicting evidence regarding efficacy and concerns about the safety of specific nutrients, such as arginine in critically ill patients, that have been associated with sepsis and acute respiratory distress syndrome, highlight the need for cautious application and further research [94]. Current evidence remains heterogeneous, and standardized protocols regarding formulation, timing, and route of administration are still lacking. Addressing these challenges through patient-centered strategies, economic evaluations, standardized guidelines, and rigorous clinical studies will be critical for optimizing the role of immunonutrition in surgical and oncologic care.

**Figure 3:**
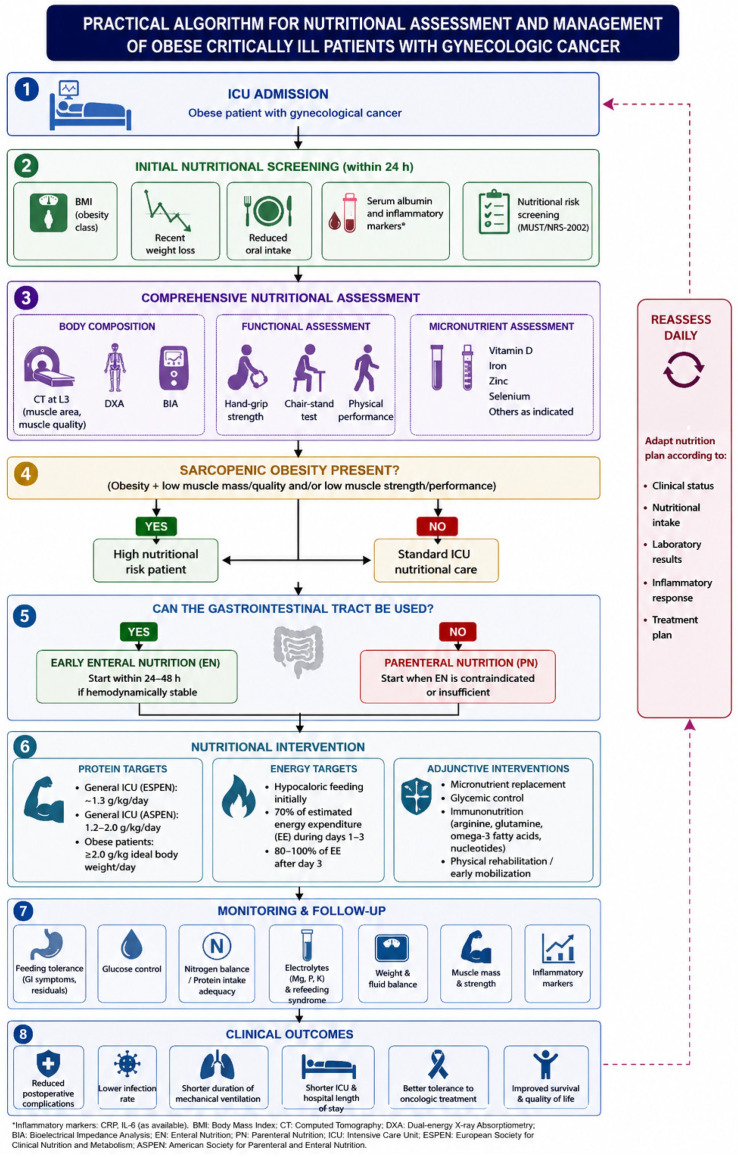
Practical algorithm for nutritional assessment and management of obese critically ill patients with gynecologic cancer.

Figure 3 presents a proposed clinical algorithm for the nutritional assessment and management of obese critically ill gynecologic oncology patients, emphasizing early risk stratification, individualized nutritional support, and continuous reassessment throughout ICU hospitalization.

## 7. Conclusions and Future Directions

Obese critically ill patients with gynecological cancer represent a uniquely vulnerable population, in whom excess adiposity conceals profound malnutrition, sarcopenia, and micronutrient deficiencies. Systemic inflammation, insulin resistance, and tumor-driven catabolism create a hypermetabolic state that complicates nutritional support, increases susceptibility to infections, delays wound healing, and heightens chemotherapy-related toxicity. Nutritional assessment should extend beyond traditional anthropometric and biochemical markers to incorporate functional measures and body composition analyses, such as CT, DXA, and bioelectrical impedance.

The limited availability of ICU-specific nutritional studies in gynecologic oncology necessitates cautious extrapolation from broader oncology and critical care literature, highlighting the need for disease-specific prospective trials. Enteral nutrition remains the preferred approach when feasible, supporting gut integrity and immune function, while parenteral nutrition serves as a critical adjunct in cases of gastrointestinal dysfunction or obstruction. High-protein regimens, hypocaloric energy provision, and supplementation of micronutrients—including vitamin D, zinc, selenium, and iron—are key components of tailored nutritional strategies. Immunonutrition, incorporating arginine, glutamine, omega-3 fatty acids, and nucleotides, may offer additional benefits by modulating inflammation, supporting immune competence, and enhancing tissue repair, although further evidence is required to define optimal formulations and timing in this population (Table 4).

This narrative review is also subject to limitations inherent to its design. The literature search was restricted to selected databases and English-language publications, which may have introduced publication bias and limited inclusion of regional data. Although major biomedical and nutrition sources were captured, relevant studies indexed exclusively in databases such as EMBASE or CINAHL may have been missed. Future systematic reviews incorporating broader database coverage are warranted to further validate and expand these findings. In addition, no formal risk-of-bias assessment or evidence grading methodology was performed, which limits the ability to quantitatively evaluate the strength and certainty of the included evidence. Furthermore, ICU-specific nutritional studies in gynecologic oncology remain scarce, necessitating cautious extrapolation from broader oncology and critical care literature.

Future research should focus on large-scale, prospective studies evaluating personalized nutritional interventions that integrate metabolic, functional, and body composition metrics. Development of standardized protocols for early identification of sarcopenic obesity, micronutrient deficiencies, and hypercatabolic states is essential. Important research priorities include defining optimal energy and protein targets in obese critically ill gynecologic oncology patients, evaluating body composition-guided nutritional strategies, clarifying the timing and composition of immunonutrition, and developing standardized protocols for early identification and management of sarcopenic obesity and micronutrient deficiencies in ICU settings. Additionally, clinical trials assessing the efficacy and safety of immunonutrition and protein-focused strategies in obese critically ill gynecological cancer patients are needed to optimize outcomes, reduce treatment-related complications, and improve survival and quality of life. Finally, emerging anti-obesity therapies, including GLP-1 receptor agonists and dual incretin agents, may further influence nutritional status in obese cancer patients. Although these agents improve metabolic control and weight reduction, concerns have emerged regarding potential loss of lean body mass, appetite suppression, and exacerbation of sarcopenia, particularly in critically ill or frail oncology patients.

## Figures and Tables

**Table 1:** Diagnosis of sarcopenic obesity based on the ESPEN-EASO consensus.

Diagnostic Tool	Details	Comments/Key Considerations
**Skeletal Muscle Function**		
**Hand-Grip Strength**	Caucasian men: <27 kg Caucasian women: <16 kg Asian men: <26 kg Asian women: <18 kg	Critical for assessing upper body strength. Cut-offs should be adjusted for age, sex, and ethnicity.
**Chair Stand Test**	5-time sit-to-stand: >15 s 30 s chair stand: Reduced repetitions	Assesses lower limb strength and endurance. Requires population-specific adjustments; less commonly used but valuable for comprehensive assessments.
**Knee Extensor Strength**	Adjusted for body mass where data are available	Gold standard due to high accuracy. Recommended for routine use where available.
**Body Composition Analysis**		
**Dual-Energy X-ray Absorptiometry (DXA)**	Measures ALM, fat mass, and skeletal muscle mass. Cut-offs: ALM/W: <7% (men), <6% (women)	Gold standard.
**Bioelectrical Impedance Analysis (BIA)**	Measures fat mass and lean mass; less precise than DXA.	Accessible and affordable alternative. May have limitations in individuals with severe obesity or altered hydration status.
**Computed Tomography (CT)**	Quantifies muscle cross-sectional area at L3 vertebra. Cut-offs: Skeletal muscle index: <52.4 cm^2^/m^2^ (men), <38.5 cm^2^/m^2^ (women)	Highly precise; ideal for oncology patients already undergoing CT scans. Not suitable for routine diagnosis due to cost and limited access.

ALM: Appendicular lean mass, CT: Computed tomography, DXA: Dual-Energy X-ray Absorptiometry, W: Weight. Diagnostic criteria are derived from ESPEN–EASO consensus statements and are not specific to gynecologic oncology populations but are commonly applied in oncologic and critical care settings [43].

**Table 2:** Clinical consequences of malnutrition in obese patients with gynecologic cancer.

	Clinical Consequences	Primary Evidence Population	Supporting Evidence
**Wound Healing**	Impaired protein synthesis → delayed wound closure, wound dehiscence, and higher rates of surgical site infection.	Gynecological cancer patients	[49]
**Immune System**	Protein-energy malnutrition worsens innate and adaptive immune responses; obesity-related inflammation amplifies dysfunction, leading to higher postoperative infection risk.	Gynecological cancer patients	[50,51,52]
**Hospitalization Burden**	Increased nosocomial infections, prolonged length of stay, higher costs, and more frequent readmissions.	Non-gynecologic patients †	[53,54,55]
**Respiratory Function**	Reduced respiratory muscle mass → difficulty weaning from ventilation, prolonged ICU and hospital stays.	Gynecological cancer patients	[56]
**Chemotherapy Tolerance**	Increased drug toxicity due to high BSA-based dosing and reduced lean body mass; higher risk of treatment modifications, delays, and interruptions.	Gynecological cancer patients	[57,58]
**Survival outcomes**	Sarcopenic obesity associated with poorer overall survival (OS) and progression-free survival (PFS); higher prevalence of postoperative complications.	Gynecological cancer patients Postoperative patients †	[56,59,60]

Findings are derived from gynecologic oncology populations where available; otherwise, data from mixed oncology cohorts are contextualized to gynecologic cancer patients. †: Evidence derived from non-gynecologic oncology populations and extrapolated based on shared pathophysiological mechanisms.

**Table 3:** Key evidence supporting nutritional management in obese critically ill patients with gynecologic cancer.

Author, Year	Population (*n*)	Design	Population/Cancer Type	Setting	Nutrition/Body Composition Focus	Key Outcome	Evidence Context
Bhasin et al., 2020 [41]	226	Prospective cohort	Gynecologic cancers (mixed)	Surgical	Sarcopenia, malnutrition	Higher morbidity and mortality	Direct gynecologic; non-ICU
Dodds et al., 2014 [42]	176	Prospective cohort	Endometrial cancer	Surgical	Sarcopenic obesity	Reduced overall survival	Direct gynecologic
Donini et al., 2022 [43]	4136	Meta-analysis	Gynecologic cancers	Mixed	Sarcopenia	Worse OS and PFS	Direct synthesis; heterogeneous
Fang et al., 2021 [73]	46	Interventional	Ovarian cancer	Surgical	Immunonutrition	Improved postoperative recovery	Direct gynecologic; non-ICU
Jiang et al., 2022 [61]	263	Observational	Gynecologic cancers	Hospital	Parenteral nutrition	Improved nutritional markers	Direct gynecologic; indirect outcomes
Jiang et al., 2025 [56]	NA	Meta-analysis	Critically ill adults	ICU	EN vs. PN	Supports EN when feasible	Indirect ICU evidence
Sehouli et al., 2021 [59]	NA	Guideline	Critically ill adults	ICU	Energy/protein targets in obesity	High-protein, hypocaloric feeding in obese population	Indirect guideline evidence
Tajchman et al., 2016 [74]	40	Validation study	Obese cancer patients	ICU	Energy requirements	ASPEN hypocaloric targets validated	Indirect ICU oncology evidence

OS, overall survival; PFS, progression-free survival; EN, enteral nutrition; PN, parenteral nutrition; ICU, intensive care unit. Selective study-level evidence directly supporting key statements in the manuscript. Studies are grouped to distinguish gynecologic cancer-specific data from indirect evidence derived from mixed oncology or ICU populations.

**Table 4:** Comparison of enteral nutrition (EN), parenteral nutrition (PN), and immunonutrition (IN) in obese critically ill gynecological cancer patients.

Feature	Enteral Nutrition (EN)	Parenteral Nutrition (PN)	Immunonutrition (IN)
**Definition/Use**	Nutrient delivery via the gastrointestinal tract Preferred when gut function is preserved	Intravenous nutrient delivery Used when EN is insufficient or contraindicated	Targeted nutrition with immunomodulatory substrates Modulates immune and inflammatory responses
**Primary Benefits**	Preserves gut integrity and mucosal barrier Reduces bacterial translocation Supports immune function	Ensures adequate nutrient delivery Maintains nitrogen balance Corrects protein-energy and micronutrient deficiencies	Reduces inflammation Enhances wound healing Mitigates muscle catabolism
**Energy Provision**	12–25 kcal/kg/day	12–25 kcal/kg/day Hypocaloric strategies in obesity	Delivered via specialized EN or PN formulations Adjusted to metabolic demands
**Advantages**	Physiological feeding route Maintains gut barrier Lower infection risk	Bypasses gastrointestinal tract Highly customizable composition	Immunomodulatory effects Potential reduction in infectious complications
**Challenges/Risks**	Feeding intolerance and delayed gastric emptying Aspiration risk Anastomotic complications	Hyperglycemia and hypertriglyceridemia Electrolyte disturbances Central line-associated infection	Higher cost Variable patient compliance Lack of standardized protocols
**Timing**	Early initiation (within 24–48 h of ICU admission or postoperatively)	Supplemental PN after ~6 days if EN inadequate Earlier initiation when EN not feasible	Ideally initiated preoperatively (5–7 days) and continued peri-/postoperatively
**Special Considerations in Obese Gynecologic Cancer Patients**	Hypocaloric, high-protein feeding Continuous or post-pyloric administration for tolerance	High protein dosing (≥2 g/kg ideal body weight) Careful glucose and lipid monitoring	Support of immune function in hyperinflammatory states Potential benefit in sarcopenic obesity
**Primary Evidence Source**	ICU and mixed oncology populations	ICU and surgical oncology populations	Predominantly surgical oncology; limited gynecologic- and ICU-specific data

Evidence for immunonutrition is largely derived from surgical oncology populations, with limited ICU- and gynecologic-specific data.

## Data Availability

No new data were created or analyzed in this study.
